# Immunogenicity of DNA, mRNA and Subunit Vaccines Against Beak and Feather Disease Virus

**DOI:** 10.3390/vaccines13070762

**Published:** 2025-07-17

**Authors:** Buyani Ndlovu, Albertha R. van Zyl, Dirk Verwoerd, Edward P. Rybicki, Inga I. Hitzeroth

**Affiliations:** 1 Biopharming Research Unit, Department of Molecular and Cell Biology, University of Cape Town, Rondebosch, Cape Town 7701, South Africa; ndljos005@myuct.ac.za (B.N.); ed.rybicki@uct.ac.za (E.P.R.); inga.hitzeroth@uct.ac.za (I.I.H.); 2 Centre for Bioprocess Engineering Research, Department of Chemical Engineering, University of Cape Town, Rondebosch, Cape Town 7701, South Africa; 3 Parrot Breeding Solutions (PTY) Ltd., Nigel 1491, South Africa; dirkv@karanbeef.com; 4 Institute of Infectious Disease and Molecular Medicine, Faculty of Health Sciences, University of Cape Town, Observatory, Cape Town 7925, South Africa

**Keywords:** mRNA vaccine, subunit vaccine, DNA vaccine, BFDV

## Abstract

Background/Objectives: Beak and feather disease virus (BFDV) is the causative agent of psittacine beak and feather disease (PBFD), affecting psittacine birds. There is currently no commercial vaccine or treatment for this disease. This study developed a novel BFDV coat protein mRNA vaccine encapsidated by TMV coat protein to form pseudovirions (PsVs) and tested its immunogenicity alongside BFDV coat protein (CP) subunit and DNA vaccine candidates. Methods: mRNA and BFDV CP subunit vaccine candidates were produced in *Nicotiana benthamiana* and subsequently purified using PEG precipitation and gradient ultracentrifugation, respectively. The DNA vaccine candidate was produced in *E. coli* cells harbouring a plasmid with a BFDV1.1mer pseudogenome. Immunogenicity of the vaccine candidates was evaluated in African grey parrot chicks. Results: Successful purification of TMV PsVs harbouring the mRNA vaccine, and of the BFDV-CP subunit vaccine, was confirmed by SDS-PAGE and western blot analysis. TEM analyses confirmed formation of TMV PsVs, while RT-PCR and RT-qPCR cDNA amplification confirmed encapsidation of the mRNA vaccine candidate within TMV particles. Restriction digests verified presence of the BFDV1.1mer genome in the plasmid. Four groups of 5 ten-week-old African grey parrot (*Psittacus erithacus*) chicks were vaccinated and received two boost vaccinations 2 weeks apart. Blood samples were collected from all four groups on day 14, 28 and 42, and sera were analysed using indirect ELISA, which showed that all vaccine candidates successfully elicited specific anti-BFDV-CP immune responses. The subunit vaccine candidate showed the strongest immune response, indicated by higher binding titres (>6400), followed by the mRNA and DNA vaccine candidates. Conclusions: The candidate vaccines present an important milestone in the search for a protective vaccine against PBFD, and their inexpensive manufacture could considerably aid commercial vaccine development.

## 1. Introduction

Beak and feather disease virus (BFDV) is the causative agent of psittacine beak and feather disease (PBFD), which affects both wild and captive birds in many parts of the world [1,2]. Infection is characterised by severe deformities in beaks and claws, dystrophic feathers and immunosuppression, which increases susceptibility to secondary infections, resulting in significant morbidity and mortality [1,3,4,5,6,7]. BFDV is a small non-enveloped icosahedral virus with virions ranging from 10 to 23 nm that belongs to the genus *Circovirus* in the *Circoviridae* family [1,4,8,9,10]. It has a circular ssDNA genome of 1.7–2 kb in size [4], encoding a replication-associated protein (Rep) and a capsid protein (CP) [11,12]. Psittacine birds are its natural hosts, but susceptibility to infection and severity of disease symptoms varies from species to species [1]. BFDV infections have also been reported to spread to non-psittacine bird species [1,13,14], including wild laughing kookaburras (*Dacelo novaeguineae*), wild sacred kingfisher (*Todiramphus sanctus*), wild tawny frogmouths (*Podargus strigoides*), a southern boobook (*Ninox boobook*), a barn owl (*Tyto alba*) and a brown goshawk (*Accipiter fasciatus*) [1]. The disease is a global concern as it causes significant mortality and potentially the extinction of susceptible endangered species [15,16], such as the orange-bellied parrot (*Neophema chrysogaster*) in Australia [17], Echo parakeet (*Psittacula echo*) in Mauritius [18], Red-fronted Parakeet (*Cyanoramphus novaezelandiae*) in New Zealand [19], Cape parrot (*Poicephalus robustus*) and black-cheeked lovebird (*Agapornis nigrigenis*) in South Africa [20]. In 2005, the Australian national government declared it a “key threatening process to biodiversity” [15].

BFDV cannot be propagated in vitro, which has hindered the development of commercial vaccines [21,22,23]; thus, there is no commercially available vaccine to protect birds against BFDV infection [1,24]. It has however been reported that inactivated BFDV isolated from the feathers of chronically infected birds was effective in immunising healthy birds against the virus [25]. Besides the lack of in vitro platforms to confirm virus inactivation, another problem with this strategy is that it relies on harvesting feathers and tissues from infected birds: this is not sustainable, as amounts and availability may be severely limiting. There are also ethical implications with keeping infected birds with the aim to harvest the virus [2]. Given the challenge of not being able to propagate the virus in tissue culture, the risk associated with the use of partially inactivated virus as well as the ethical implications for keeping infected birds, recombinant DNA technologies have become a promising alternative for the development of BFDV vaccines [9].

BFDV CP has been recombinantly expressed in a number of expression platforms including bacteria [26,27], yeast [28], insect cells [29,30] and plants [9,24,31] as a strategy to develop subunit vaccine candidates. The BFDV CP recombinantly produced using the baculovirus expression system was immunogenic when tested in *Cacatua tenuirostris* [2]. Vaccinated birds showed reduced virus replication and had no clinical symptoms compared to non-vaccinated birds. The authors noted, however, that the vaccination regime needed optimisation to protect the young chicks, which are more susceptible to PBFD. These results indicated that BFDV CP could be an ideal vaccine candidate to protect birds from PBFD subject to further dosage optimisation [2]. However, since then there have been no published studies that tested the immunogenicity of BFDV CP in birds. Thus, there remains a need to develop vaccines to protect psittacine bird species from BFDV. The urgency is from both conservation and economic standpoints, as parrots are among endangered bird species, and parrot breeders suffer significant financial losses from the disease.

Our laboratory has for some time been involved in expressing BFDV CP in both insect cells [32] and plants, with successes that include showing that virus-like particles (VLPs) constituted solely of BFDV CP can be made and purified from plants [9,24,31,33]. However, yields were low and scale up of production would have been difficult given a dependence on density gradient centrifugation [9,24,31,33].

In the last two decades, there has been a shift from traditional virus- and protein-based vaccines to nucleic acid-based vaccines [34,35,36], particularly towards the development of mRNA vaccines. The mRNA vaccines developed for SARS-CoV-2 proved to be highly successful with several advantages over traditional approaches [35,36]. These showed highly effective protective immunity, a high level of safety and the potential for rapid and highly scalable production, and they had fewer systemic side effects [35,36]. A few studies have been foundational in exploring mRNA as a vaccine platform [35,36]. One involved the direct injection of naked in vitro transcribed (IVT) mRNA into mouse skeletal muscle, which resulted in the expression of reporter genes within the muscle cells [37]. In another study, liposome-enclosed IVT mRNA encoding influenza virus nucleoprotein elicited virus-specific cytotoxic T lymphocytes in mice [38]. Yet another study injected mice with luciferase mRNA and carcinoembryonic antigen mRNA to elicit production of antigen-specific antibodies [39]. Taken together, these studies demonstrated that mRNA elicits both cellular and humoral immune responses in the host. This makes mRNA vaccines promising candidates for prevention and treatment of infectious diseases and cancers [35,36,40]. The findings however did not stimulate interest in pre-COVID times to significantly invest in mRNA vaccines and therapeutics development due to concerns about mRNA instability, innate immunogenicity and its delivery and survival within the host [35].

Recent technological advancements have spiked interest in the mRNA platform as a promising tool in vaccine development and protein replacement therapy [35]. For example, recent research efforts have discovered many approaches for protecting mRNA delivered in vivo [36,41,42]. Among those is the use of different types of lipid nanoparticles (LNPs) to successfully deliver exogenous mRNA in vivo [36]. LNPs encapsulate mRNA in a shell as a result of the electrostatic interaction between the ionisable lipids and the negatively charged mRNA [43]. This protects mRNA during in vivo delivery and targets it to different cell types using various routes [36,44,45,46,47,48]. The first two COVID-19 mRNA vaccines approved by the Food and Drug Administration (FDA) [36], BNT162b2 (Pfizer/BioNTech) and mRNA-1273 (Moderna), used LNPs in their formulation for in vivo delivery and stability [49].

The major drawback with COVID-19 mRNA-LNP vaccine formulations, apart from serious reactogenicity due to the LNPs, was their required storage temperature ranging between −20 °C and −80 °C for a maximum period of three to six months [49,50]. This presented a challenge to distribute the vaccines in developing and under-developed countries as they often lack infrastructure for long-term cold storage [36]. Understanding the root cause of mRNA-LNP instability may drive innovation of stable formulations that do not require ultralow temperatures [49]. Hence many efforts have been made by various COVID-19 mRNA vaccine manufacturers and the WHO to address the storage temperature problem and to make vaccines easily accessible to the larger population of the world [36,49,50,51,52,53].

Tobacco mosaic virus (TMV) is a rodlike single-stranded RNA virus that is a pathogen to a wide range of host plants including tobacco. Native TMV particles are 300 nm in length with an outer diameter of 18 nm and inner cavity of 4 nm wide [54,55,56]. TMV capsids consist of 2130 identical coat protein (CP) subunits with a molecular weight of 17.5 kDa. These CP subunits are coiled around the 6395 bp single-stranded viral RNA [54,57]. The length of a TMV particle is determined by the length of RNA encapsidated [58]. TMV pseudovirions (PsVs) that contain foreign nucleic acids instead of viral RNA were first reported in two studies in the early 1970s and the mid-80s [59,60]. Both studies reported that plant host RNA was encapsidated within TMV particles. A large proportion of encapsidated host RNA was complementary to both tobacco chloroplast DNA and nuclear DNA. This phenomenon was later explored as a tool to deliver foreign nucleic acids to different cell types [61,62,63]. One study reported encapsidation of pre-lysozyme mRNA in TMV coat protein (CP) rods in the presence of a TMV origin of assembly (OAS) RNA sequence near the 3′ terminus of the pre-lysozyme mRNA sequence [62]. A similar study demonstrated encapsidation of mRNA encoding chloramphenicol acetyltransferase (CAT) into rodlike particles now more widely referred to as PsVs. After inoculation of plant protoplasts or intact plant leaves, TMV PsVs were uncoated, resulting in successful expression of the CAT protein [61]. The authors observed higher levels of CAT expression when mRNA was delivered encapsidated within TMV particles compared to naked delivery. The same trend was observed when *Xenopus laevis* oocytes were microinjected with TMV particles and with naked TMV RNA [63]; the authors further concluded that intact TMV particles must have disassembled to release functional RNA within the cytoplasm of the animal cells. Taken together, these studies indicated the potential of TMV PsVs as delivery system for foreign nucleic acids into different cell types. TMV particles, like most plant virus capsids, are very stable and resistant to ribonuclease activity. This protects RNA against degradation while the virus, or PsVs, accumulate within the host cells [61,62].

The present study therefore aimed to develop an mRNA vaccine candidate against BFDV that is relatively inexpensive to produce, stable, safe to use and easy to distribute, with the potential for long-term storage between 2 °C and 8 °C. To achieve this, mRNA encoding BFDV capsid protein (CP) was proposed to be enclosed within TMV rods through transient expression of TMV CP in *Nicotiana benthamiana* (*N. benthamiana*) leaves, by co-infiltration with a recombinant BFDV CP mRNA construct fused with the TMV origin of assembly (OAS) RNA sequence near the 3′ terminus. Immunogenicity of this novel vaccine candidate was to be tested in African grey parrot chicks and compared to two other vaccine candidates, namely, an *E. coli*-produced synthetic infectious BFDV DNA vaccine [64] and plant-produced BFDV CP subunit vaccine. The African grey parrot species is susceptible to BFDV and therefore it is a perfect model to test immunogenicity of candidate vaccines.

## 2. Materials and Methods

### 2.1. Growth Media, Bacterial Strains, Restriction Enzymes and PCR Reagents

All *Escherichia coli* (*E. coli*) and *Agrobacterium tumefaciens* strains were grown in Luria Bertani (LB) liquid media and/or on solid agar plates supplemented with appropriate antibiotics. Antibiotics were purchased from SIGMA-Aldrich, Burlington, MA, USA. DH5-a chemically competent *E. coli* cells (New England Biolabs, Ipswich, MA, USA) or Stellar chemically competent cells (TaKaRa Bio Group, San Jose, CA, USA) were used for *E. coli* transformations with ligation reactions, while *Agrobacterium*-mediated transformations were all performed using *Agrobacterium* strain GV3101::pMP90RK electrocompetent cells. Seeds (LAB line) for growing *N. benthamiana* plants were purchased from Herbalistics Pty Ltd., Queensland, Australia.

Restriction enzymes used in this study were purchased from Thermo Fisher Scientific, Waltham, MA, USA and New England Biolabs, Ipswich, MA, USA. All PCR reactions were performed using Taq DNA polymerase master mix red (Ampliqon, Odense, Denmark) or any of the following: Phusion High-Fidelity PCR Master Mix, Phusion Flash High-Fidelity PCR Master Mix and Phusion Hot Start II High-Fidelity DNA Polymerase, all from Thermo Fisher Scientific, Waltham, MA, USA. High-Fidelity Taq was used for cloning or creating new constructs and Ampliqon Taq used for screening of recombinant colonies.

### 2.2. Cloning and Transformation of E. coli and Agrobacterium

**Figure 1:**
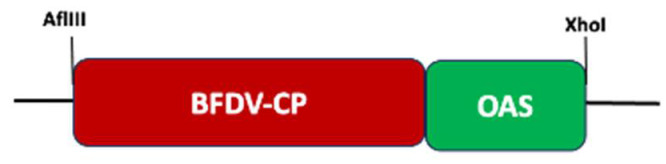
Schematic diagram showing Budgerigar BFDV coat protein and TMV origin of assembly fusion (BFDV-CP-OAS). The flanking restriction sites were used for cloning into pRIC4, a replicating plant expression vector (diagram not drawn to scale).

The BFDV CP gene construct (Figure 1) was created using the *cp* sequence from BFDV isolate BKS1ZA_84 (Genbank accession number GQ165756) obtained from Budgerigar [65]. The online platform from TaKaRa Bio (https://www.takarabio.com/learning-centers/cloning/primer-design-and-other-tools, accessed on 11 October 2021) was used to design primers for In-Fusion cloning. The BFDV *cp* gene (753 bp) sequence was PCR-amplified from the pRIC3-BFDV-CP [9] by using the Oligo1-BFDV-CP-Fwd/Oligo2-BFDV-CP-Rev primer pair (Table 1), amplified DNA was gel-purified with the E.Z.N.A. Gel Extraction Kit (Omega Bio-tek, Inc., Norcross, Georgia) according to the manufacturer’s instructions. The TMV-OAS sequence was amplified from the pUC57-Mini-AGP-CP-OAS plasmid DNA using Oligo3-OriA-Fwd and Oligo4-OriA-Rev and the PCR product was gel-purified as described above. The purified BFDV *cp* and OAS PCR fragments were fused into a linearised (*Afl*III/*Xho*I) pRIC4 vector using In-Fusion^®^ HD Cloning (TaKaRa Bio, Paris, France). The fusion reaction was transformed into Stellar chemically competent cells following manufacturer’s protocol and plated on to LB agar supplemented with 100 mg/mL ampicillin. Colonies were screened by PCR using the pTRA-Fwd/pTRA-Rev primer pair (Table 1) and PCR products were separated on 0.8% agarose gels stained with ethidium bromide at 120 V. Plasmid DNA was isolated from positive colonies using the QIAprep Spin Miniprep Kit (Qiagen, Hilden, Germany) and sent for sequencing (Macrogen, Amsterdam, The Netherlands).

Following confirmation by PCR and sequencing, plasmid DNA was transformed into GV3101::pMP90RK electrocompetent *Agrobacterium* cells through electroporation [66]. After incubation, cells were concentrated by centrifugation in a benchtop centrifuge and resuspended in 100 mL LB broth before platting out on LB agar plates supplemented with 50 mg/mL carbenicillin, 30 mg/mL kanamycin and 50 mg/mL rifampicin. Plates were incubated at 27 °C for 2–3 days, after which colonies were screened by PCR (pTRA-Fwd/pTRA-Rev).

### 2.3. Agrobacterium Infiltration of N. benthamiana

Ten millilitres (mL) of LB broth supplemented with 30 mg kanamycin, 50 mg rifampicin and 25 mg carbenicillin were each inoculated with 2 mL glycerol stocks of *Agrobacterium* cultures for pRIC4-BFDV-CP-OAS and pRIC4-TMV-CP. These cultures were incubated overnight at 27 °C with agitation. A volume of 10 mL of the overnight cultures were inoculated into 50 mL LBB broth media supplemented with the antibiotics and grown overnight at 27 °C with agitation. These cultures were further upscaled to 500 mL culture volumes and in addition to antibiotics 20 mM acetosyringone (Merck Life Science (PTY) Ltd., Johannesburg, South Africa) was also added to the cultures, and cultures were incubated as above. Cultures were diluted in induction media (10 mM MES, 10 mM MgCl_2_, pH 5.6) to an optical density (OD) of 1:1 for each culture. *N. benthamiana* plants were grown for 4–5 weeks at 22 °C under 16-h light and 8-h dark cycles. Sixty *N. benthamiana* plants (4–5 weeks old) were co-infiltrated with TMV-CP/BFDV-CP-OAS cultures using a vacuum infiltrator. After infiltration, plants were kept under the same growth conditions and harvested seven days post infiltration (dpi).

For a subunit vaccine production, plants were infiltrated with recombinant pRIC-BFDV-CP *Agrobacterium* at an OD of 0.5. As a negative control, plants were infiltrated with an *Agrobacterium* culture harbouring the pRIC vector lacking an insert, and leaves were harvested four days post infiltration.

### 2.4. Purification of TMV PsVs

TMV PsVs were purified according to the protocol described [67] with modifications. In summary, leaves were harvested, fresh biomass weighed and homogenised in 2 volumes (*w*/*v*) of sodium acetate (NaAc) buffer (50 mM sodium acetate, 0.86 M NaCl (5% *w*/*v*), pH 5) with or without Complete^TM^ EDTA-free protease inhibitor cocktail (Roche, Basel, Switzerland). Leaf homogenates were filtered through 4 layers of Miracloth (Merck) before measuring the pH. If the pH of the homogenates was above 5, then it was adjusted with concentrated H_3_PO_4_. After adjusting the pH to 5, clarified homogenates were incubated at 60 °C for 15 min with occasional swirling. This was done to facilitate removal of RuBisCO. Homogenates were cooled down at 4 °C for 15 min and thereafter centrifuged at 6000× *g* for 10 min at 4 °C. Clarified supernatants were transferred into fresh glass beakers or Erlenmeyer flasks, then 4% polyethylene glycol (PEG) MW 8000 was added and the mixture was incubated overnight at 4 °C. PEG precipitates were centrifuged at 10,000× *g* for 10 min and pellets resuspended in a small volume of PBS (pH 7.4) buffer. PEG-purified samples were transferred into sterile tubes and stored at 4 °C. To confirm sample purity from bacterial contamination, PEG-purified TMV/BFDV-CP-OAS samples were streaked on LB media lacking antibiotics and incubated overnight at 37 °C, and plates were examined for any bacterial growth.

### 2.5. Purification of a BFDV CP Subunit Vaccine Candidate

For sucrose cushion ultracentrifugation, biomass was harvested (75 g) and homogenised in DB150 buffer (150 mM NaCl, 1 mM CaCl_2_, 0.001% Triton X-100, 0.25 M L-Arginine, 10% glycerol (*v*/*v*), 10 mM Tris/HCL (pH 6.5)) with the addition of 1× Complete EDTA-free Protease Inhibitor (Roche). The crude sample was clarified at 25,931× *g* for 30 min at 4 °C and supernatants filtered through 4 layers of Miracloth^™^ (Merck, Kenilworth, NJ, USA). Sucrose cushions were prepared in Thinwall 38 mL Ulta-Clear^TM^ ultracentrifuge tubes (Beckman Coulter, Inc., Placentia, CA, USA) by underlaying 6 mL of 25% and 2 mL of 70% sucrose made in DB150 buffer. Tubes were loaded with 30 mL of filtered plant juice and centrifuged at 4 °C for 4 h in a Beckman SW32Ti rotor at 175,000× *g*, after which fractions were collected from the bottom of the tubes. Fractions from 25% and 70% sucrose were combined and dialysed overnight in 1× PBS (Sigma-Aldrich). Dialysed samples were centrifuged at 13,000 rpm for 20 min to remove dialysis buffer and to concentrate samples.

To further purify protein samples, iodixanol density gradient ultracentrifugation was performed. Solutions of iodixanol at 20%, 30%, 40% and 50% were prepared and gradients were prepared by underlaying the iodixanol steps beneath the concentrated extract. Ultracentrifugation was performed at 175,000× *g* for 4 h at 4 °C. Following centrifugation, 1–1.5 mL fractions were collected from the bottom of the tubes and stored at −20 °C until further analysis. Aliquots of the pooled fractions were streaked on LB agar plates to confirm sample purity from bacterial contamination prior to vaccination of birds.

### 2.6. SDS-PAGE, Coomassie Blue Staining, Protein Quantification and Western Blot Analysis

Crude and PEG-purified TMV/BFDV-CP-OAS PsVs samples were denatured in 5× SAB at 95 °C for 10 min and separated in 15% SDS-PAGE gels for 2 h at 120 V. Filtered and heated samples were also separated as described above together with BSA standards (15–0.47 mg). After separation, gels were incubated in Coomassie Brilliant Blue R-250 (Sigma-Aldrich) (1% Coomassie blue stock (62.5 mL), methanol (250 mL), glacial acetic acid (80 mL) and water (127 mL) stain solution for an hour at 37 °C with shaking. This was followed by rinsing a few times with distilled water before destaining with methanol/glacial acetic acid (30% methanol/10% glacial acetic acid/60% H_2_O) solution at room temperature with shaking. Gel images were scanned and protein band intensity quantified using Gene Tools version 3.07.03 software from Syngene. BSA standards were plotted against their corresponding gel densitometry values to create a standard curve. The BSA standard curve was subsequently used to calculate the relative TMV CP concentration of the PsVs.

For plant-produced BFDV CP, gradient-purified fractions were denatured, analysed on 12% SDS-PAGE gels and gel densitometry used to quantify BFDV-CP relative to a BSA standard as described in Section 2.6 above. For western blot analysis, fractions were pooled, resolved on 12% SDS-PAGE gels and transferred onto Hybond^TM^ C Extra nitrocellulose membranes (AEC—Amersham, Midrand, South Africa) using a Transblot^®^ SD semi-dry transfer cell (Bio-Rad, Irvine, CA, USA) at 15 V for 1 h 30 min. PageRuler™ Plus Prestained Protein Ladder (Thermo Fischer Scientific, Waltham, MA, USA) was used as size marker and to indicate successful protein transfer onto nitrocellulose membranes. Following transfer, the membranes were incubated in freshly made blocking buffer (1× PBS (pH 7.4), 5% long life fat-free milk, 0.1% Tween 20) for 30 min at room temperature with shaking. After blocking, rabbit-raised anti-BFDV sera (1:2000) diluted in blocking buffer was added to the membranes and incubated at 4 °C overnight with shaking. Following incubation, the membranes were washed 4× 15 min in blocking buffer with shaking at room temperature. Following the wash steps, membranes were probed with anti-rabbit IgG alkaline phosphatase-conjugated (1:10,000, Sigma) secondary antibody diluted in blocking buffer at 37 °C for 60 min with shaking. Membranes were washed 4× 15 min in blocking buffer without milk at room temperature with shaking, after which 3–5 mL of 5-bromo-4-chloro-3-inodyl phosphate (BCIP) and nitroblue tetrazolium (NTB) phosphatase substrate (BCIP/NBT 1-component, KPL) was added for approximately an hour to develop the membranes. Reactions were stopped by rinsing membranes in water when bands had developed sufficiently.

### 2.7. Transmission Electron Microscopy (TEM) to Confirm Presence of TMV PsVs

To confirm the presence of TMV rods following co-expression of TMV CP with BFDV-CP-OAS, PEG-precipitated samples were either filter sterilised (0.45 µM) or heated at 70 °C for 20 min to remove any bacterial contamination. Sterilised and heat-treated samples were trapped on copper grids and analysed with TEM. Briefly, protein samples were analysed for presence of TMV rods using a FEI Tecnai F20 transmission electron microscope. To this end, carbon coated copper grids (mesh size 200) were made hydrophilic by glow discharging at 25 mA for 30 s using a Model 900 SmartSet Cold Stage Controller (Electron Microscopy Sciences, Hatfield, South Africa). Carbon-coated grids were placed on 20 mL samples and incubated at room temperature for 5 min. This was followed by 5× washes by placing grids on 10 mL drops of distilled water and blotting off excess water in between washes. Grids were negatively stained by floating on 20 mL drops of 2% uranyl acetate for 1 min. Excess uranyl acetate was blotted off using filter paper. Grids were stored at room temperature until viewed under TEM.

### 2.8. RNA Extraction and RT-PCR to Confirm BFDV-CP mRNA Encapsidation into TMV Rods

One hundred and fifty µL of PEG-purified TMV PsV-containing samples were treated with 63 units (0.25 mL) of Benzonase^®^ Nuclease (Merk Life Science (PTY) Ltd., Johannesburg, South Africa) for 24 h at room temperature. The samples were heated for 30 min at 70 °C to inactivate Benzonase and allowed to cool down to room temperature. RNA was extracted using the QIAamp^®^ Viral RNA Mini kit (QIAGEN, Hilden, Germany) following the manufacturer’s protocol, after which the extracted RNA was treated with DNase (Promega, Madison, WI, USA) according to the manufacturer’s protocol. Five to eight microliters of freshly extracted RNA was used per reaction and the volume was adjusted accordingly with RNase-free water. If the recommended amount of DNase was not sufficient to remove all DNA in the sample, then the amount added was increased incrementally. To confirm if DNase treatment was successful, PCR was conducted using the treated RNA as template and gene specific primer pair (Oligo1-BFDV-CP-Fwd/Oligo2-BFDV-CP-Rev) (Table 1).

First-strand cDNA synthesis was performed using M-MLV Reverse Transcriptase from Promega following manufacturer’s protocol. In summary, 8–12 mL DNA-free RNA was added to 0.5 mg (0.73 mL of a 100 mM stock) Oligo dT20 Anchored primer and heated at 70 °C for 5 min to melt any secondary structures within the template. The rest of the steps were followed as specified in the Promega Usage Information sheet. Synthesised cDNA was used immediately or stored at −20 °C until needed.

To confirm BFDV-CP-OAS encapsidation, PCR reactions were performed using a BFDV-CP-specific primer pair (Oligo1-BFDV-CP-Fwd/Oligo2-BFDV-CP-Rev), and RNA (untreated), RNA (DNase treated) and cDNA were used as templates. PCR products were analysed on 0.8% agarose gel stained with ethidium bromide.

### 2.9. Real-Time qPCR (RT-qPCR) to Quantify Encapsidated mRNA

DNase-treated RNA samples extracted from 140 µL (A), 280 µL (B) and 560 µL (C) of purified TMV/BFDV-CP-OAS PsVs were sent to Inqaba Biotech (Pretoria, South Africa) to perform One-step RT-qPCR using a Luna^®^ Universal One-Step RT-qPCR Kit (New England Biolabs). In summary, one microliter (1 µL) of a 1:1000 dilution of each of RNA sample (A, B and C) was used as template for one-step qPCR using SYBR Green, reactions were carried out in triplicate. The ΔΔCT method was used to calculate the differential expression of BFDV CP in sample B and C using sample A as a control.

Statistical analyses were performed using GraphPad Prism 10 software. Statistical significance was calculated between the three sample groups (A, B and C) using the Ordinary one-way Analysis of Variance (Ordinary one-way ANOVA) and Tukey’s multiple comparisons test to determine significance (*p* < 0.05).

### 2.10. Large-Scale Production and Isolation of BFDV 1.1mer (DNA Vaccine Candidate)

The BFDV 1.1mer is a synthetic molecular DNA clone based on BFDV isolate BKS1ZA_84 (Genbank accession number GQ165756) obtained from budgerigars in South Africa [65]. The double stranded DNA genome was synthesised in silico by GeneArt as previously described [64]. This was initially designed to be used as a challenge model in budgerigars.

The BFDV 1.1mer, maintained in the pMA cloning vector (GeneArt, Thermo Fisher Scientific), was grown in 2× 500 mL LB broth media supplemented with ampicillin (100 ug/mL). Plasmid DNA was isolated using the EndoFree^®^ Plasmid Mega Kit from QIAGEN according to the manufacturer’s instructions. Purified plasmid DNA was digested using different combinations of restriction enzymes (*Pst*I/*Hind*III, *Pst*I/*Xba*I, *Stu*I/*Hind*III and *Stu*I/*Xba*I) to confirm integrity of the clones, the clones were also sent for sequence analysis with primers specific to the *rep* and *cp* genes.

### 2.11. Immunisation of African Grey Parrots

The current study was approved by the Science Faculty Animal Ethics Committee at the University of Cape Town (2022/V12/IH), Faculty of Science Biological Safety Committee (FSBC-2022-003-IH), South African Health Products Regulatory Authority (SAHPRA, VCT 04/2022) and by the Department of Agriculture, Land Reform and Rural Development (Section 20 reference number: 12/11/1/7/3 (2438 AC)). Ten-week-old African grey parrot (*Psittacus erithacus*) chicks were recruited by Dr Verwoerd from Parrot Breeding Solutions (PBS) at Wyksrus Quarantine station in Nigel, Gauteng. Blood samples were collected from all the chicks prior the start of the immunisation trial to test for BFDV infection using PCR (SMT Veterinary Laboratory, Centurion, South Africa). Birds were acclimatised for 7 days prior to vaccination and were randomly allocated into four groups of five birds each (Table 2). The three candidate vaccines tested included the BFDV mRNA vaccine, BFDV CP subunit vaccine and the BFDV 1.1mer DNA vaccine candidate. A fourth group of birds were vaccinated with PBS and served as negative control in this study.

**Figure 2:**
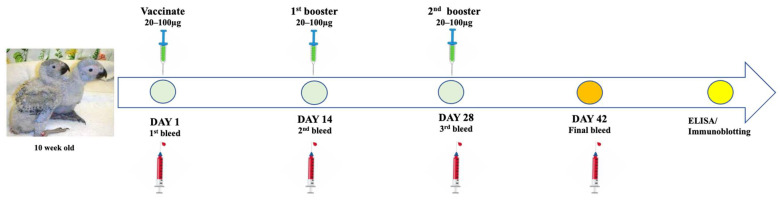
Illustration of vaccine administration and blood collection from the African grey parrot chicks throughout the trial of the study.

On day 1 of the study, chicks were weighed using a scale and the bodyweight of each bird recorded (Table S1). Blood samples were collected from the leg vein using a 23G needle prior to vaccination. Birds from each vaccine group were subcutaneously injected with the respective antigen on the side of the breast using a 23G needle. Figure 2 below illustrates vaccination administration and blood sampling throughout the trial.

On day 14 and 28, blood samples were collected from all 4 groups and birds received the second and third doses of their respective vaccine candidates. Throughout the study the birds were monitored daily for development of adverse conditions. On day 42, a final bleed was performed by drawing 1–2 mL of blood from each bird in all 4 groups, blood was kept at 4 °C. Birds were monitored for adverse reactions or illness before being released to integrate with the rest of the birds on the farm. Sera were harvested from blood samples and kept at 4 °C before shipping to the Biopharming Research Unit at the University of Cape Town. Sera were analysed for antigen-specific antibodies using ELISA and immunoblotting assays.

### 2.12. Humoral Immune Response Analysis and Determination of Binding Titres Using Indirect ELISA

Ninety-six-well Maxisorp Nunc-Immuno Plates (Thermo Fisher Scientific, Odense, Denmark) were coated with 100 µL (100 ng) per well of plant-produced BFDV CP and pRIC (−VE) antigens diluted in coating buffer (10 mM Tris pH 8.5) and incubated overnight at 4 °C with gently shaking. Following that, plates were blocked with 200 µL of 1× TBS blocking buffer (500 mM Tris, 1.5 M NaCl, pH 7.5, with 5% non-fat dry milk) for 1 h at 37 °C with shaking. After blocking, 100 µL of 1:100 dilution of respective antisera were added as primary antibodies and plates incubated for one hour at 37 °C with shaking. Plates were washed 4× with 200 µL 1× TBS pH 7.5 (with 0.05% Tween-20) after incubation and subsequently probed with anti-bird secondary antibody (1:5000) for one hour at 37 °C with shaking. After incubation, plates were washed 4× with 200 µL of 1× TBS buffer (500 mM Tris, 1.5 M NaCl, pH 9) and one hundred (100 µL) KPL ABTS 1-Component Peroxidase Substrate was added to the wells and plates were incubated for 30 min in the dark, followed by absorbance measurement at 405 nm using a Multiskan Go Spectrophotometer (Thermo Fisher Scientific, Ratastie 2, Finland). Average absorbance values from the negative control antigen wells were subtracted from the experimental average absorbance values.

To determine binding titres of the final bleed, sera from 5 birds in each group were pooled and dilutions thereof (1:100–1:6400) were used as primary antibodies in an indirect ELISA. The binding titres for each group were expressed as the reciprocal of maximum dilution that elicited an immune response with absorbance readings higher than that of the PBS control serum diluted 1:100.

### 2.13. Statistical Analysis

Statistical analyses were performed using GraphPad Prism 10 software. Statistical significance was calculated between the three vaccine groups (DNA, BFDV CP subunit and mRNA vaccine) and PBS negative control group using the Two-way Repeated Measures Analysis of Variance (Two-way RM ANOVA) and Tukey’s multiple comparisons test to determine significance (*p* < 0.05).

## 3. Results

### 3.1. Transformation of BFDV-CP-OAS into E. coli and Agrobacterium Competent Cells

The BFDV CP gene sequence, isolate BKS1ZA_84 (Genbank accession number GQ165756) from budgerigars, was used to create BFDV-CP-OAS construct. The BFDV-CP and OAS sequences were amplified from their parental plasmids (pRIC3-BFDV-CP and pRIC4-EGFP-SARS-conc-OAS), fused into one fragment using assembly PCR and successfully ligated into replicating plant-expression vector (pRIC4) before transforming into *E. coli* and *Agrobacterium* competent cells, respectively (Figures S1 and S2).

### 3.2. Co-Expression of TMV-CP with BFDV-CP-OAS

**Figure 3:**
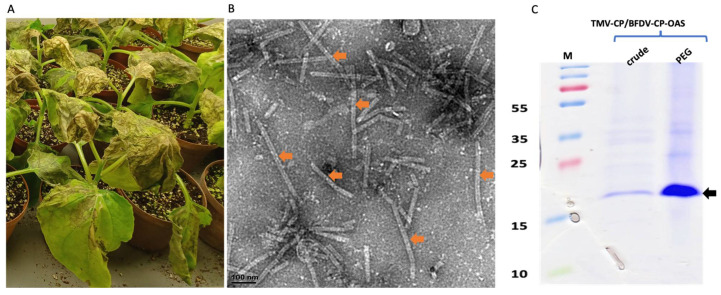
Expression confirmation of TMV rods encapsidating BFDV-CP-OAS mRNA. Images showing leaves that were harvested 7 dpi when they were very necrotic (**A**). TEM image showing TMV rods of various sizes, ranging from 50 to 350 nm (indicated by orange arrows). The expected TMV/BFDV-CP-OAS is approximately 89 nm in length. The scale bar is 100 nm. (**B**). Scale bar indicates 100 nm (**B**). Coomassie stained gel image of TMV-CP expression in *N. benthamiana* leaves harvested on 7 dpi (**C**). Crude: crude samples, PEG: 4% PEG-precipitated samples. PageRuler™ Prestained Protein Ladder Plus in kDa (Thermo Fisher Scientific). Black arrow indicates the size of TMV-CP, approximately 17.5 kDa.

The BFDV-CP-OAS construct was co-infiltrated with TMV-CP into *N. benthamiana* leaves at an OD ratio of 1:1. Leaves were harvested at 7 dpi when plants had become necrotic (Figure 3A), and protein was extracted and precipitated using 4% PEG. Crude and precipitated samples were analysed with TEM and on SDS-PAGE gels. TEM analyses revealed successful rod formation for both crude and PEG-purified samples showing presence of a mixed-rod-size population (shown in orange arrow) ranging from 50 to 350 nm (Figure 3B). Based on the BFDV-CP transcript size, the expected rod size for TMV/BFDV-CP-OAS was 89 nm. No difference was observed in TMV rods abundance between crude and precipitated samples. Both crude and PEG precipitated samples gave a distinct TMV coat protein band size of approximately 17.5 kDa (shown in black arrow), and as expected, the band observed for the precipitated samples were more intense compared to the band seen in the crude sample (Figure 3C).

### 3.3. TEM Analyses, SDS-PAGE Separation and Quantification of Filter-Sterilised and Heat-Treated Samples

**Figure 4:**
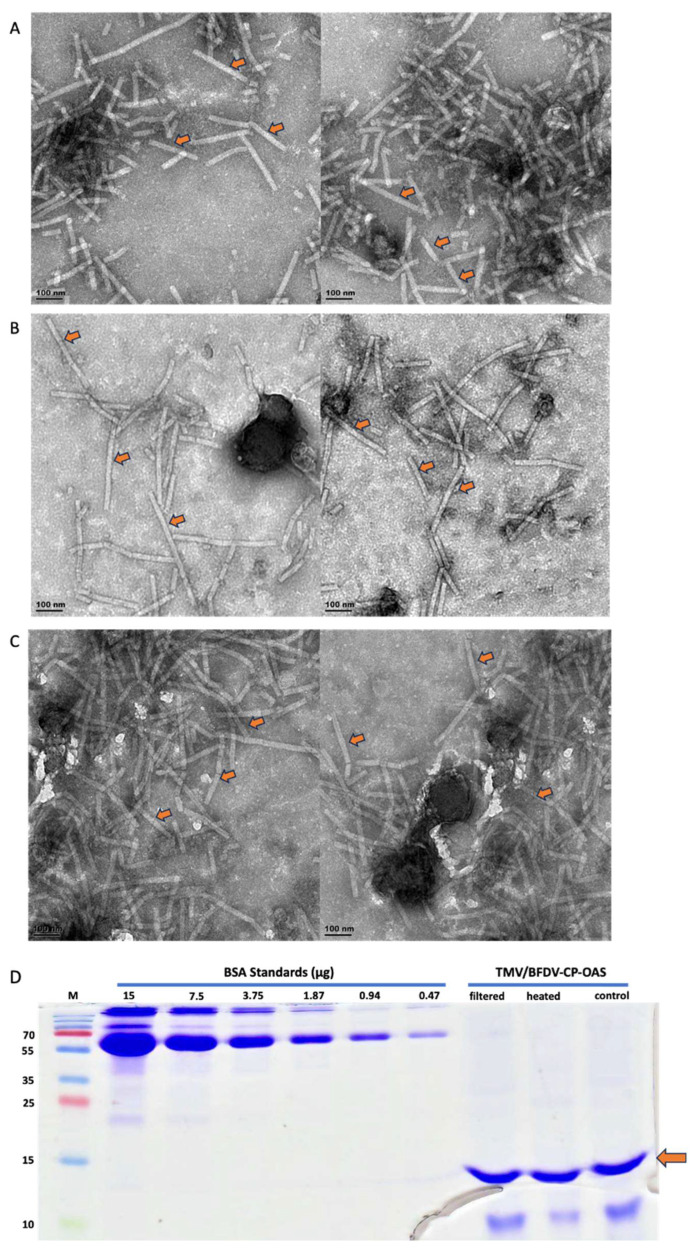
TEM analysis of TMV/BFDV-CP-OAS extractions prepared from leaf material harvested 7 dpi and purified using PEG precipitation. These are representative images indicating that neither filter-sterilisation or heat-treatment had any negative effects on the abundance and integrity of TMV rods. (**A**) filtered sample, (**B**) heated sample, (**C**) control sample. Arrows indicate sample TMV rods. Scale bar indicates 100 nm. (**D**) Quantification of TMV/BFDV-CP-OAS samples using BSA standards ranging from 15 to 0.47 µg. Samples were either filtered using 0.45 µM syringe filter (filtered), heated at 70 °C for 20 min (heated), or not treated at all (control). A band of approximately 15 kDA was observed in both the treated and control samples, as indicated by the orange arrow. M: PageRuler^TM^ Prestained Protein Ladder Plus (Thermo Fischer Scientific).

To confirm that filter sterilisation and heat treatment did not affect the stability and integrity of the TMV rods, TEM analysis was carried out on the treated and control samples. TEM analysis showed no difference in the abundance and integrity of particles observed in both treatments. Figure 4A–C shows representative images of PsVs obtained from treatments samples and a control sample. TMV particles encapsidating BFDV-CP-OAS were expected to be 89 nm in length. However, rods of various lengths were obtained.

The same treated samples were analysed on a 15% SDS-PAGE. TMV-CP bands of approximately 15 kDa (Figure 4D), which was slightly below the expected 17.5 kDa were detected for all samples. It is common for proteins to migrate slightly below or above their expected size. The band intensity of filtered, heat-treated and control samples looked similar indicating that none of the treatments had negative effects. BSA standards were used to quantify protein samples and the values obtained were similar (0.256 mg/mL, 0.251 mg/mL and 0.257 mg/mL, respectively) for all three samples (Figure 4D).

### 3.4. PCR and One-Step RT-qPCR Confirmed Encapsidation of Nucleic Acids into TMV Particles

**Figure 5:**
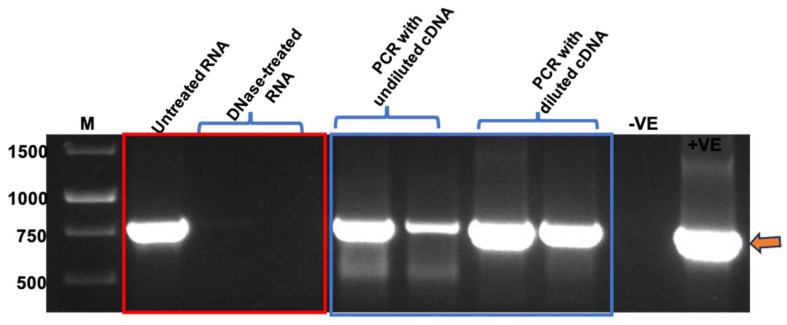
PCR using RNA (in red rectangle) and cDNA (in blue rectangle) samples as template. GeneRuler^TM^ 1 kb DNA Ladder in bp (M), No template control (−VE), Plasmid DNA as control (+VE). RNA samples were treated with two different concentrations of DNase to evaluate the most effective concentration. First strand of cDNA was synthesised using both RNA treatments.

After confirming the presence and integrity of TMV rods, PCR and RT-qPCR were performed to confirm the packaging of BFDV-CP mRNA within TMV rods. Prior to RNA extraction, TMV PsVs were treated with Benzonase to degrade all nucleic acids not encapsidated within the TMV rods. Following nucleic acid extraction, RNA samples were treated with DNase to remove any DNA contamination present in the samples. This was necessary as the QIAamp^®^ Viral RNA Mini kit (QIAGEN) extracts both RNA and DNA from TMV PsVs samples. When RNA samples not treated with DNase were used as PCR template, a fragment corresponding to BFDV *cp* gene (~750 bp) was detected (Figure 5), indicating DNA presence in the RNA samples. This was in line with our previous observations of intense PCR bands detected at approximately 750 bp when TMV rods were not treated with Benzonase prior to RNA extraction, indicating the presence of nucleic acids both within and on the outside of the PsVs. Complete treatment of extracted RNA samples with DNase resulted in no PCR product (Figure 5), indicating that only RNA was present in the sample. RNA that gave no PCR product after DNase treatment was used to synthesise first strand cDNA. It was anticipated that undiluted cDNA may contain reagents that could interfere with the PCR reaction, therefore both diluted and undiluted samples were used as template. PCR products of approximately 750 bp corresponding to the BFDV *cp* gene were obtained in all reactions where cDNA was used as template (Figure 5), indicating that BFDV CP mRNA was encapsidated within TMV rods.

Our initial RT-qPCR results confirmed that mRNA was packaged within TMV particles (Table S2). However absolute copy numbers did not increase relative to the amount of TMV-CP used for RNA extraction (Appendix A). In addition, mRNA copy numbers also varied greatly between RNA extractions from the same sample at different occasions, indicating that sample handling during preparation of RNA, DNase treatment and cDNA synthesis may have contributed to variability within the RNA used for RT-qPCR. To determine if this was indeed the case, one-step RT-qPCR was performed instead of a two-step RT-qPCR. One-step RT-qPCR allows cDNA synthesis and real-time PCR in a single reaction tube and thus minimises sample handling that may introduce errors and contamination. RNA was extracted from 140 µL (A), 280 µL (B) and 560 µL (C) of purified TMV PsVs sample, prior to qPCR, a conventional PCR was carried out to confirm complete DNase treatment of the samples. No PCR product was detected in the PCR reactions where treated RNA was used as template, while in all reactions where RNA not treated with DNase was used, a product corresponding to BFDV-CP gene was detected (Figure S3).

**Figure 6:**
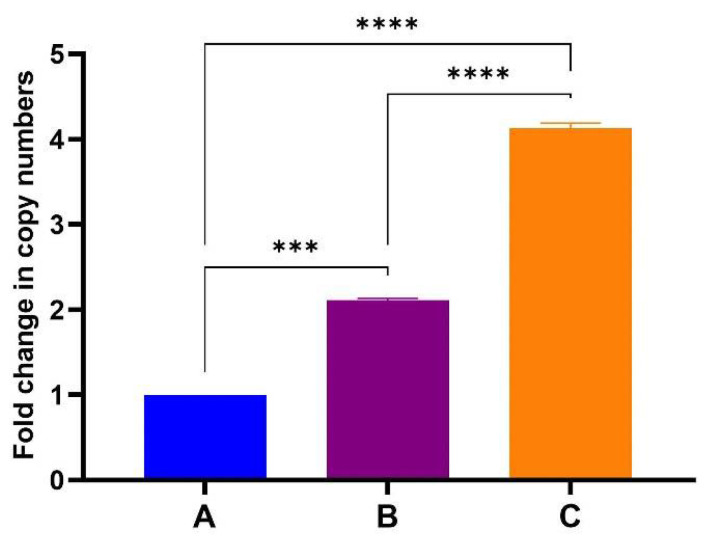
Fold change in BFDV-CP gene copy numbers between samples A, B and C. Average Cq values of two individual experiments in triplicates using RNA samples from a single extraction. The ΔΔCT method was used to quantify the differential expression of BFDV CP in samples B and C, using sample A as the reference control. Error bars represent standard error of means. The BFDV-CP mRNA copies obtained in sample B and C were statistically significantly higher (*p* = 0.0005 and *p* < 0.0001) compared to the control sample A (denoted by *** and ****). The mRNA copies in sample C were also significantly higher (*p* < 0.0001) compared to sample B (denoted by ****).

After confirming that the samples did not contain any DNA, one-step RT-qPCR analysis were performed by Inqaba Biotechnical Industries (Pty) Ltd., Pretoria, South Africa. One-step RT-qPCR showed a proportionate increase in gene copy numbers with increasing amount of TMV-CP sample used for RNA extraction (Figure 6). These reactions were run in duplicate with no significant variation in the data obtained as indicated by standard deviation error bars. The BFDV-CP mRNA copies obtained in sample B is about double as in sample A and sample C is again double of B. This corresponds to the amount of TMV that the mRNA was extracted from. When qPCR was repeated (three times and in triplicates) with freshly extracted RNA on each occasion, a slight variation was observed between data sets (Figure S4). The mRNA copies in sample B were not double that of A while C was more than 4-fold that of A. Despite that slight variation the data were consistent in that mRNA copy numbers increased with the amount of TMV-CP sample used for RNA extraction. Establishing that mRNA levels increase in proportion to TMV-CP will be important in optimising the vaccine dosage since it is TMV-CP (PsVs) that will be used for vaccination.

### 3.5. Production of the BFDV-CP Subunit Vaccine Candidate

**Figure 7:**
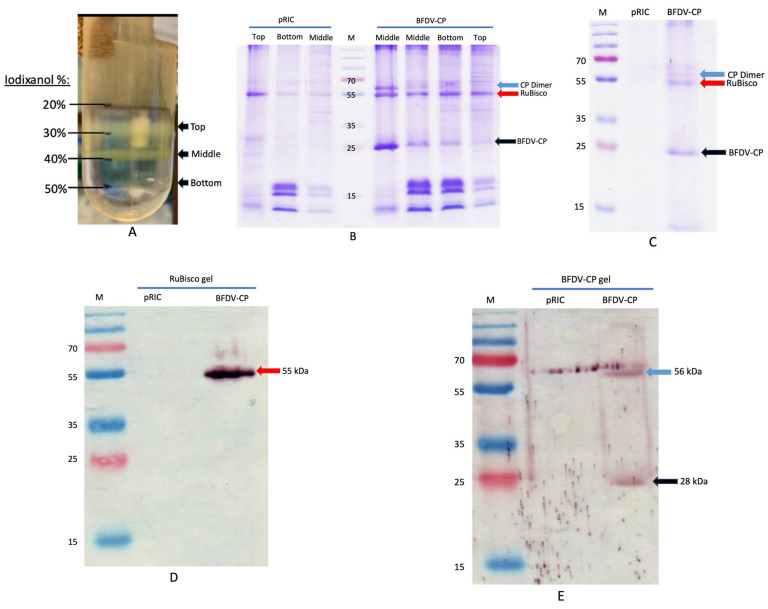
(**A**–**E**). Image of a Thinwall 38 mL Ulta-Clear^TM^ ultracentrifuge tube showing different fractions (top, middle, bottom bands) after iodixanol (20–50%) density gradient ultracentrifugation of concentrated extract at 175,000× *g* for 4 h at 4 °C (**A**). SDS-PAGE and western blot analyses of plant-produced BFDV CP (**B**–**E**). pRIC: negative control, empty plant expression vector; BFDV-CP: pRIC with BFDV CP; Lane M: protein ladder; (**B**) Fractions from density gradient; Middle (Fraction collected at the middle band; Bottom (fraction collected at the bottom band; Top (fraction collected at the top band). Black arrow = BFDV-CP of 28 kDa and blue arrow = BFDV-CP dimer of 56 kDa. Red arrow = plant RuBisCO of 55 kDa. (**C**) pooled samples from gradient. Blot (**D**) was probed with 1: 5000 commercial rabbit anti-RuBisCO primary antibody and 1:10,000 anti-rabbit IgG AP-conjugated for the secondary antibody. Blot (**E**) was probed with 1:2000 anti BFDV CP sera (#6841 final) for primary antibody and 1:10,000 anti-rabbit IgG AP-conjugated for secondary antibody.

Plant-produced BFDV CP was purified using two-step gradient ultracentrifugation. After centrifugation, fractions were collected between 20 and 50% iodixanol (top, middle and bottom bands) (Figure 7A) and analysed on SDS-Page. A clear band at ~28 kDa (indicated with a black arrow) was detected in all fractions collected after BFDV-CP purification (Figure 7B). This band was not present in the negative control (pRIC) samples (Figure 7B). An additional band was detected for BFDV-CP at ~56 kDa, indicating the presence of a dimer (Figure 7B). Bands constituting RuBisCO (~55 kDa), a highly abundant plant protein, was also detected in all the fractions (top, middle and bottom bands) in the purified samples (Figure 7B). All fractions from plant-purified BFDV-CP were pooled and analysed again on the Coomassie gel and the same banding pattern was detected (Figure 7C). To quantify BFDV-CP, gel densitometry was carried out where the amount of CP was determined relative to BSA standards of known concentration. To further confirm the identity of the bands detected on the Coomassie-stained SDS-Page gels, western blot analysis was carried out to specifically detect RuBisCO and the BFDV-CP dimer. Both proteins were detected in the purified BFDV-CP fractions, but not in the negative control pRIC samples (Figure 7D,E), indicating that RuBisCO was co-purified with BFDV-CP.

### 3.6. Production of the BFDV 1.1mer DNA Vaccine Candidate

The pMA-BFDV1.1mer plasmid DNA was isolated and quantified using a Nanodrop spectrophotometer. The constructs were evaluated and confirmed with restriction enzyme digests and sequencing.

### 3.7. Immunogenicity of the BFDV Vaccine Candidates in African Grey Parrot Chicks

#### 3.7.1. Pre-Immunisation PCR to Confirm Absence of BFDV Infection

PCR screening confirmed that all chicks tested negative for BFDV infection (Supplementary File), indicating they had no prior exposure to BFDV. Therefore, these birds were ideal candidates for testing the antigens in this study. Four groups of 5 birds each were vaccinated on day 1, 14 and 28. Blood samples were collected from all 4 groups on day 14, 28 and 42 and sera were analysed.

#### 3.7.2. Indirect ELISA Analysis for Antigen Specific Antibodies and Determination of Binding Titres

**Figure 8:**
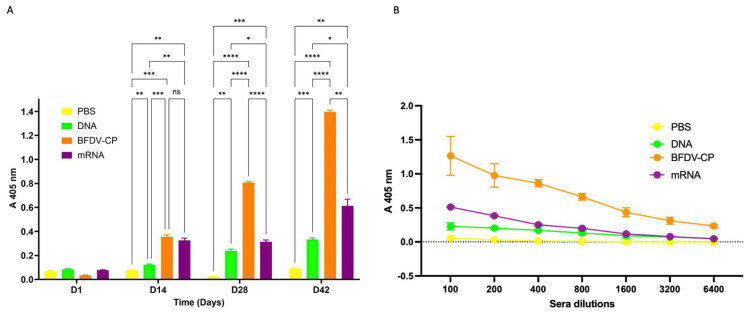
(**A**) Indirect ELISA assay analysing the immune response induced by sera samples from candidate vaccines and PBS control. Antigen: plant-purified BFDV-CP; primary antibody: pooled sera (DAY 1, 14, 28 and 42) from respective vaccine groups (1:100); secondary antibody: anti-bird (1:5000). Data represent averages from four individual experiments (plates) with four replicates in each experiment. Error bars represent standard error of means. Statistical significance was calculated using the two-way RM ANOVA and *p*-values were calculated based on Tukey’s multiple comparisons test with 95% Cl. Asterisk (*) denotes statistical significance. **D14**: All three vaccine candidate groups (DNA, BFDV-CP and mRNA) elicited statistically higher (*p* = 0.0074, *p* = 0.0007 and *p* = 0.0016) immune response compared to negative control (denoted by **, *** and ** respectively). BFDV-CP and mRNA vaccines induced statistically higher immune response (*p* = 0.0008 and *p* = 0.0024) compared to DNA vaccine (denoted by *** and ** respectively). There was no statistically significant difference (*p* = 0.6743, denoted by ns) between the immune response induced by BFDV-CP (A 405 nm = 0.354) and mRNA vaccine (A 405 nm = 0.325). **D28**: the three vaccines, DNA, BFDV-CP and mRNA maintained significantly higher (*p* = 0.0016, *p* < 0.0001 and *p* = 0.0008) immune responses compared to the PBS control (denoted by **, ****, and *** respectively). BFDV-CP and mRNA vaccines elicited 3.4- and 1.3-fold higher immune response (*p* < 0.0001 and *p* = 0.0458) compared to the DNA vaccine (denoted by **** and *), and BFDV-CP was 2.6-fold higher (*p* < 0.0001) compared to mRNA vaccine (denoted by ****). **D42**: DNA, BFDV-CP and mRNA vaccines still had a much higher immune response (*p* = 0.0001, *p* < 0.0001 and *p* = 0.0073) compared to PBS control (denoted by ***, **** and **). BFDV-CP and mRNA vaccine immune response had increased to 4.2- and 1.8-fold higher (*p* < 0.0001 and *p* = 0.0421) compared to DNA vaccine (denoted by **** and *), and BFDV-CP was 2.3-fold higher (*p* = 0.0016) than mRNA vaccine (denoted by **). (**B**) Titrations of sera from four vaccine groups: PBS (negative control), DNA, BFDV-CP and mRNA group. Antigen: plant-purified BFDV-CP; primary antibody: pooled sera (DAY 1, 14, 28 and 42) from respective vaccine groups (1:100–1:6400); secondary antibody: anti-bird (1:5000). Data represent averages from two individual experiments (plates) with three replicates in each experiment. Error bars represent standard error of means.

Indirect ELISAs with plant-produced BFDV-CP were performed to generate quantitative and decisive immunogenicity data compared to western and dot blot assays. ELISAs showed that sera from the PBS control and Day 1 bleeds of all vaccine groups elicited no anti-BFDV immune response, with an A 405 nm of 0.08 and less on average (Figure 8A).

Sera analysed from day 14 showed that the three vaccine candidates, DNA, BFDV CP and mRNA elicited immune responses that were 1.6-, 4.7- and 4.3-fold higher when compared to the PBS negative control vaccine group (Figure 8A). This indicated that all three vaccine candidate groups elicited statistically higher (*p* = 0.0074, *p* = 0.0007 and *p =* 0.0016) immune responses compared to the negative control. Comparing immune responses induced between the three candidate vaccines, the BFDV CP and mRNA vaccine candidates elicited 2.9- and 2.7-fold higher immune responses compared to the DNA vaccine candidate, these responses were statistically significant (*p* = 0.0008 and *p* = 0.0024). On the other hand, there was no statistically significant difference (*p* = 0.6743) between the immune responses induced by the BFDV CP (A 405 nm = 0.354) and mRNA vaccine candidates (A 405 nm = 0.325).

At day 28, the three vaccine candidates maintained significantly higher (*p* = 0.0016, *p* < 0.0001 and *p* = 0.0008) titres compared to the PBS control (Figure 8A). BFDV-CP and mRNA vaccines elicited 3.4- and 1.3-fold higher immune response (*p* < 0.0001 and *p* = 0.0458) compared to the DNA vaccine, and BFDV-CP was 2.6-fold higher (*p* < 0.0001) compared to mRNA vaccine (Figure 8A). This indicates that BFDV CP elicited the strongest immune response that increased from statistically not significant (on day 14) to 2.6-fold higher compared to the mRNA vaccine candidate.

For day 42 sera analysed, a similar trend in the immune response induced (observed in day 28) was observed between the four vaccine groups. DNA, BFDV-CP and mRNA vaccines still had a much higher immune response (*p* = 0.0001, *p* < 0.0001 and *p* = 0.0073) compared to PBS control (Figure 8A). BFDV-CP and mRNA vaccine immune response had increased to 4.2- and 1.8-fold higher (*p* < 0.0001 and *p* = 0.0421) compared to DNA vaccine, and BFDV-CP was 2.3-fold higher (*p* = 0.0016) than mRNA vaccine. BFDV-CP vaccine induced strongest immune response in both day 28 and 42 sera samples.

Anti-BFDV-CP binding titres were also determined with indirect ELISA assay. Wells were coated with 100 ng plant-produced BFDV-CP antigen, probed with diluted sera samples and binding titres determined as the reciprocal of the maximum dilution that elicits immune response with absorbance readings higher than those of the PBS control at 1:100 dilution. The PBS control group sera elicited no binding titres against the plant produced BFDV-CP antigen, with only ~0.055 OD_405_ at 1:100 dilution (Figure 8B). On the other hand, the DNA, BFDV-CP and mRNA vaccine groups elicited anti-BFDV-CP binding titres of 800 (~0.130 OD_405_), >6400 (~0.233 OD_405_) and 1600 (~0.116 OD_405_), respectively (Figure 8B).

## 4. Discussion

Viral coat proteins are a major component of viruses with various important roles, including protecting viral nucleic acids from degradation by many factors such as hostile temperatures outside the host and a cascade of host defence mechanisms during the infection process. Virion surface structural proteins have been studied widely because they are the first point of contact with the host. They contain specific binding sites to enable recognition and attachment of the virus to specific receptor molecules on the host cell surface. This has made viral CPs the most suitable antigen and focus for vaccine development strategies, especially those based on recombinant technologies.

In this study, we report on the development of a novel mRNA vaccine candidate for BFDV and compare it to a DNA vaccine candidate and a recombinantly produced subunit vaccine candidate. The immunogenicity of these vaccine candidates was evaluated in 10-week-old African grey parrot chicks.

The mRNA vaccine was produced *in planta* and purified using 4% PEG to yield TMV particles about 0.30 µg/µL. TEM analysis revealed formation of TMV rods (Figure 3B) of varying sizes, ranging from 50 to 350 nm. RT-PCR confirmed encapsidation of both DNA and RNA into TMV rods (Figure 5). To determine the amount of mRNA copies encapsidated within TMV-CP, relative (One-Step) RT-qPCR was performed and confirmed that mRNA copies increased proportionately with the amount of purified TMV-CP protein sample used for RNA extraction (Figure 6). This is important for dosage determination since the vaccine administered was based on the amount of TMV-CP, not the actual mRNA copies encapsidated. Therefore, if vaccinating animals, for example, with 100 µg (333 µL) of PsVs, as was the case in the present study, induces a sub-optimal response, which necessitates a dosage increase, then increasing the dosage to 200 µg would mean the mRNA copies have also increased proportionately. The quantification of mRNA copies would however need to be performed for each batch of TMV-CP as the yield may differ from batch to batch. In our initial experiments, absolute quantification (using two-step RT-qPCR) of mRNA copies within the TMV-CP was inconclusive and needed further optimisation (Appendix A). However, a one-step RT-qPCR assay resulted in consistent results in different experiments. The present study has employed both qualitative (RT-PCR) and quantitative (RT-qPCR) approaches to determine mRNA encapsidation within the TMV particles. Previous studies have only focused on the qualitative assays such as denaturing agarose gels and Northern blots to determine mRNA encapsidation [68,69,70].

The subunit vaccine was produced and purified from plants using sucrose cushion ultracentrifugation and iodixanol density gradient ultracentrifugation. This yielded protein batches of ~0.1088 and ~0.1076 µg/µL that were detected using SDS-PAGE and Coomassie-stained gels and western blots (Figure 7B–E). RuBisCO is the most abundant protein in plant leaves that is involved in photosynthesis. Its abundant presence interferes with purification of recombinant proteins of interest expressed in plants. In the present study, SDS-PAGE analyses (Figure 7B,C) revealed that BFDV-CP (~56 kDa) co-purified with RuBisCO (55 kDa). This was confirmed by probing western blots (Figure 7D,E) with anti-RuBisCO and anti-BFDV CP-specific antibodies, respectively. The presence of RuBisCO protein in BFDV-CP subunit vaccine does not pose any danger since this protein is available in most plant-based foods consumed by humans and animals. Previous studies in our research group have also observed co-purification of BFDV CP with RuBisCO protein [24,64].

For the DNA vaccine candidate, recombinant *E. coli* harbouring a BFDV synthetic genome synthesised by GeneArt (pMA-BFDV1.1mer), was grown in LB medium from which large-scale plasmid DNA was purified to a yield of ~5.26 µg/µL. Restriction digests and sequencing confirmed the plasmid to be correct. BFDV1.1mer is an infectious molecular clone that was initially designed to be used as a challenge model to test the efficacy of BFDV candidate vaccines [64,71]. To be used for this purpose, it needed to be functional at the level of DNA replication and protein expression. In 2015, Regnard [64] investigated replication of this BFDV molecular DNA clone in vitro by transfecting HEK293TT cells, and also *in planta*, by cloning it into a plant expression vector for transient expression in *N. benthamiana* leaves. In both instances, RCA and qPCR analyses revealed replication of BDFV DNA. As a follow-up study, Buyse [33] in 2022 transfected QM7 quail tissue culture cells with plasmid DNA of the same molecular DNA clone designed by Regnard (2015) [64]. RCA and qPCR analyses revealed replication of synthetic BFDV DNA in these cell lines, while western blots confirmed expression of BFDV-CP [33]. Both these studies indicated that the BFDV1.1mer can potentially be used as a challenge model in animal studies, eliminating the need for harvesting live virus from deceased or euthanised parrots [33]. However, in the present study, BFDV1.1mer was used as a vaccine candidate. The rationale was that since the BFDV isolate BKS1ZA_84 was obtained from budgerigars; it was not expected to cause disease in the African grey parrots.

The three vaccine candidates were used to vaccinate 10-week-old African grey parrot chicks at day 1, 14 and 28, and PBS was used as a negative control. Blood samples were collected on day 1 (pre-vaccination), 14, 28 and final bleed on day 42. Sera were harvested from five birds per treatment group at each time point and pooled prior to analysis. Individual analysis of sera samples would have provided more detailed immunological data and enabled robust statistical evaluation. However, pooling was performed to ensure sufficient sample volume for all planned assays and to optimise the use of time and resources.

These were analysed by indirect ELISA using plant produced BFDV-CP as coating antigen and probed with anti-bird secondary antibodies. No anti-BFDV responses were detected with sera from the PBS vaccine group and sera from day 1 (pre-immunisation) of all the vaccine groups. On the other hand, sera from three vaccine groups elicited a stronger immune response compared to PBS and day 1 (pre-vaccination) sera (Figure 8A). The BFDV CP subunit vaccine was most immunogenic of all three as shown by higher antigen binding titres (>6400) compared to mRNA (1600) and DNA (800) vaccines (Figure 8B).

The BFDV CP subunit vaccine candidate was highly immunogenic compared to the mRNA and DNA vaccines. It is probable that the protein subunit was presented as a ready antigen to the immune system and in the right conformation to be readily taken up by antigen-presenting cells, while on the other hand, mRNA and DNA vaccines firstly required transcription and translation into antigens. It is also possible that some CP subunits assembled into VLPs, which are highly immunogenic and elicit both humoral and cellular immune responses [9], and thus resulted in much stronger immunogenicity. In the case of the mRNA vaccine candidate, dose-dependent studies would be useful to ascertain at what dose maximal immunogenicity is obtained. A different study investigated immunogenicity of E1 and E2 spike proteins from salmonid alphavirus 3 and made similar observations. The study compared protective immunogenicity of E1/E2 protein subunit vaccines with corresponding DNA vaccines against lethal challenge of the virus in salmon fish. Subunit vaccines gave better immune response and protection against mortality, while DNA vaccines showed marginal immune response and could not protect against mortality and viral replication [72]. However, inactivated whole virus, which was their positive control, conferred superior immune response over both subunit and DNA vaccines and protected against disease development in the internal organs. Another study obtained a similar result on the immunogenicity of DNA and subunit candidate vaccines based on BFDV CP gene in budgerigars (*Melopsittacus undulatus*). Both vaccine candidates elicited BFDV specific antibodies, with a subunit candidate inducing stronger immune response [73].

DNA vaccines are easier and more cost effective to produce than protein-based vaccines; as they do not require the same level of downstream processing, are very stable and do not require cold storage [74,75]. However, the only drawback is their weak immunogenic response [75,76]. It is therefore not surprising that our DNA vaccine elicited a weaker immune response than the subunit vaccine candidate. DNA vaccines induce humoral and cellular immune response, including cytotoxic T-lymphocyte (CTL) responses without posing any safety concerns as it is the case with live virus vaccines [77,78,79]. Studies have demonstrated that vaccines which induce CD8 T-cells only were capable of fully protecting the host from viral diseases without inducing any virus-specific antibodies [79,80,81,82,83,84]. To determine immunogenicity of the DNA vaccine candidate, the present study focused on antibody responses, which are easier to detect compared to T-cell response [79]. It is therefore possible that the DNA vaccine evaluated in this study elicited a protective CTL response, although confirming this was beyond the scope and timeline of the present study.

Several approaches have been explored to improve DNA vaccination, these include the use of adjuvants and alternative delivery devices [75]. Some studies have used chemical adjuvants with DNA vaccines but without investigating the actual effect of adjuvants [85,86,87,88]. Therefore, it has not been conclusive whether they improve immunogenicity of vaccines or not. The present study used Polygen, a polymer-based adjuvant (MVP Adjuvants, Omaha), which is claimed to work well with inactivated or modified live viral antigens or with subunit vaccines (mvpadjuvants.com/PHIB-17018-Adjuvants-Bulletin_Polygen). However, this adjuvant might not have been ideal for the DNA vaccine in the present study.

Another strategy that could boost immunogenicity of DNA vaccines is the use of replicating DNA vector systems from different sources [34]. Our research group has previously demonstrated that the BFDV genome could be replicated in mammalian cells with the aid of a geminivirus-based replicating vector system [71]. Similar studies have been done for other circoviruses, where attenuated molecular clones derived from porcine circoviruses 1 and 2 (PCV-1 and PCV-2) replicated in mammalian cells and caused milder infection lesions compared to parental infectious clone (PCV-2), when injected into pigs. These also induced specific (PCV-1/PCV-2) antibodies in pigs [89]. In the present study, the DNA vaccine based on the BFDV genome did not cause any symptoms of infection in African grey parrot chicks, yet it managed to induce BFDV specific antibodies. This could be because the isolate was not from African grey parrots, hence it was attenuated—or that a non-natural mode of “infection” (injection into muscle) led to a very different response. Hence this could be an ideal vaccine to protect these birds against BFDV, pending challenge with an infectious viral isolate obtained from African grey parrots.

The mRNA vaccine candidate developed in this study is potentially the most attractive option. This is because COVID-19 mRNA vaccines have successful and have put RNA based vaccines in the spotlight due to the advantages they have over DNA-based vaccines, protein subunit vaccines and other more traditional approaches [35,36]. Amongst the many advantages of using RNA platforms is safety. RNA is not infectious; it does not integrate into the host cell genome and has short in vivo half-life compared to DNA [35]. Previous studies [61,62,63] have motivated our rationale for developing the BFDV mRNA vaccine candidate described in the current study. This vaccine described here consisted of mRNA encapsidated within TMV-CP rods. Following injection of African grey parrot chicks with a total of 100 µg TMV particles per dose, the vaccine successfully elicited antibodies specific to BFDV-CP (Figure 8A,B).

The exact amount of mRNA (and DNA) copies injected into the parrot chicks could not be determined, as absolute quantification by RT-qPCR ranged from 12,104,511 to 782,790,888 copies (Calculated from Table A1 and Table S2 Data). The cDNA template used was prepared from two different RNA extractions and the RT-qPCR experiments were performed on different days under similar conditions. One-step RT-qPCR in this case was sufficient to determine that mRNA copies varied from batch to batch of TMV particle purifications and that mRNA copies increased proportionately with increasing amounts of TMV-CP used for RNA extraction (Figure 6). Thus, the dose of the mRNA vaccine injected into the chicks was determined based on the amount of TMV-CP (100 µg) not the exact quantity of mRNA copies encapsidated. Optimising for much stronger immune response in this case would therefore require experimenting with varying amounts of TMV particles determined by quantifying TMV-CP sample.

RNA is known to be less stable than DNA, hence is more susceptible to be targeted and degraded by different proteins or enzymes within the cell cytoplasm [35]. It is therefore possible that in some instances RNA is degraded before it could be translated into an antigen of choice by the host cell machinery. It would however be of interest to inject the birds with different amounts of naked RNA to determine if the RNA induces immunogenicity and how it compares with the immune responses elicited by encapsidated mRNA. In preliminary experiments we transfected HEK293T cells with naked RNA encoding BFDV-CP and with mRNA encapsidated within TMV particles. Western blot analysis showed the successful expression of BFDV-CP in cells transfected with naked RNA, with no expression detected from transfected with TMV particles. These results were consistent with those obtained in another study, where transfecting the cells with naked mRNA enhanced yellow fluorescent protein (EYFP), resulted in higher protein expression compared to transfection with mRNA encapsidated within TMV rods [90]. The authors understood this as being the result of naked mRNA, which could directly be translated in the lipofections, while on the other hand, TMV-encapsidated mRNA required unloading prior translation and expression.

Few studies have shown that naked RNA delivered in vivo resulted in protein production. One involved the direct injection of mRNAs encoding a reporter gene into mouse skeletal muscle and protein expression was detected [37]. Another study injected mice with mRNA transcripts encoding luciferase and human carcinoembryonic antigens, and the result was the production of antibodies specific to each antigen [39]. However, a recent study [90] observed the opposite; injecting mice with naked EYFP mRNA resulted in no detection in draining lymph nodes (dLNs), while delivering EYFP mRNA protected within the TMV and cowpea chlorotic mottle virus (CCMV) particles resulted in strong expression. Their results demonstrated the role of virus-like particles in protecting mRNA from degrading RNases, delivering it for cell uptake and presenting it to the cell ribosomal machinery for translation and protein expression [90].

Taken together, this study produced and tested three BFDV vaccine candidates in African grey parrot chicks. All three candidates elicited specific immune responses compared to the PBS negative control. BFDV-CP and mRNA induced up to 4.2- and 1.8-fold higher immune response compared to the DNA vaccine, while BFDV-CP was up to 2.6-fold higher than the mRNA vaccine (Figure 8A). BFDV-CP subunit vaccine candidate therefore induced strongest immune response compared to DNA and mRNA vaccine. To the best of our knowledge, this is the first study to evaluate and compare these three vaccine types in African grey parrots, or possibly in any psittacine species. It is also the first study to develop and test a novel mRNA vaccine candidate against BFDV. This represents an important step toward the development of a vaccine to protect psittacine birds against BFDV, especially given that no commercial vaccine is currently available for this purpose.

Follow up studies will focus on optimising dosage of these vaccines to further improve the strength of the immune response. Once the optimal dosages have been identified, challenge experiments using live, infectious BFDV isolated from deceased parrots will be conducted to evaluate vaccine efficacy. In the case of BFDV, where no distinct serotypes have been identified and there is a high likelihood of conserved epitopes among isolates, it may be possible to develop a single vaccine from one isolate that provides broad protection against multiple virus strains [9,91]. Future studies should further explore a heterologous prime-boost vaccination strategy, using different combinations of these vaccines, to determine whether this approach enhances the immune response and provides protection against BFDV. This concept was explored during the COVID-19 pandemic, where populations were vaccinated with different vaccines targeting the same virus, resulting in an enhanced immune response [92,93,94].

## 5. Conclusions

The candidate vaccines discussed in this study present a significant milestone in search for a protective vaccine against PBFD in endangered wild and captive parrots. In particular, the use of a mRNA vaccine encapsidated in TMV coat protein and made *in planta* was both highly novel and promising in terms of possible provision of inexpensive vaccines against a serious bird disease.

## Figures and Tables

**Table 1:** Primers used for cloning, confirming clones and RT-qPCR.

Primer Name	Sequence (5′-3)	Tm (°C)	Purpose
pTRA-Fwd	CATTTCATTTGGAGAGGACACG	60	Sequencing and Colony PCR
pTRA-Rev	GAACTACTCACACATTATTCTGG	60	
Oligo1-BFDV-CP-Fwd	TATAAAAAAAAAAAAACATGTGGGGCACCTCTAACTGCG	62.38	In-Fusion cloning PCR/PCR
Oligo2-BFDV-CP-Rev	ATTTCTATAACTACCCTTCAGTTCTGGGATTATTG	59.75	
Oligo3-OriA-Fwd	TGAAGGGTAGTTATAGAAATAATATAAAATTAGG	62.44	In-Fusion cloning PCR/PCR
Oligo4-OriA-Rev	GCGGACTCTAGAGAGCTCGAGTACTACTATTTTTCCCTTTG	67.5	
BFDV_F	AGGAGCAAACGTCAAGCACTAC	54.84	qPCR
BFDV_R	GGGGCAAACTGACGGAATTGAA	54.84	

**Table 2:** Candidate vaccines groups, vaccine dosages and vaccination schedule followed.

Vaccine Group	Vaccine Doses(µg)	Vaccine/Polygen Adjuvant Ratio	No of Birds per Group
DNA Vaccine	90	1:1	5
BFDV CP Vaccine	20	1:1	5
mRNA Vaccine	100	2:1	5
Negative Control	DPBS Buffer	1:1	5

**Figure A1:**
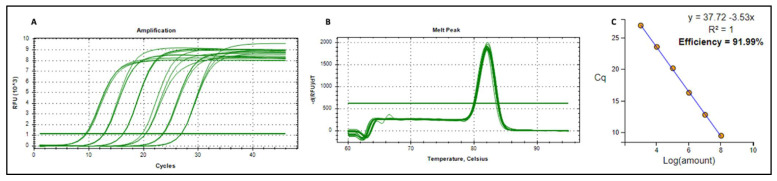
Amplification plot (**A**), melt peak (**B**) and standard curve (**C**) of pRIC4-BFDV-CP-OAS plasmid DNA.

## Data Availability

Data available on request.

## Supplementary Materials

The following supporting information can be downloaded at: https://www.mdpi.com/article/10.3390/vaccines13070762/s1, Figure S1: Creating the pRIC4-BFDV-CP-OAS construct; Figure S2: Confirmation of recombinant *Agrobacterium*; Figure S3: PCR to confirm DNase treatment of RNA samples; Figure S4: Fold change in BFDV-CP gene copy numbers between samples A, B and C; Table S1: Additional information of the African grey parrot chicks that were used in the trial; Table S2: Additional results confirming successful encapsidation of BFDV cp mRNA within TMV particles.

## Appendix A

### Amplification Plots Melt Peak and Standard Curve for pRIC4-BFDV-CP-OAS Plasmid DNA

Plasmid DNA for pRIC4-BFDV-CP-OAS was used for serial dilution (1 ng–1 fg) of standards.

Amplification plot, melt peak and standard curve indicated good PCR conditions for a target amplified (Figure A1(A–C)).

**Table A1:** RT-qPCR results quantifying BFDV-CP mRNA copy numbers from the cDNA using pRIC4-BFDV-CP-OAS plasmid DNA standards.

Target	Sample Type	Sample	Cq	Average Cq	Absolute Copies
pRIC4-BFDV-CP-OAS	Standard	1 ng	9.60	9.47	110,034,823
pRIC4-BFDV-CP-OAS	Standard	1 ng	9.45		
pRIC4-BFDV-CP-OAS	Standard	1 ng	9.35		
pRIC4-BFDV-CP-OAS	Standard	0.1 ng	12.84	12.78	11,003,482.3
pRIC4-BFDV-CP-OAS	Standard	0.1 ng	12.68		
pRIC4-BFDV-CP-OAS	Standard	0.1 ng	12.82		
pRIC4-BFDV-CP-OAS	Standard	10 pg	16.28	16.27	1,100,348.23
pRIC4-BFDV-CP-OAS	Standard	10 pg	16.29		
pRIC4-BFDV-CP-OAS	Standard	10 pg	16.23		
pRIC4-BFDV-CP-OAS	Standard	1 pg	19.72	20.15	110,034.823
pRIC4-BFDV-CP-OAS	Standard	1 pg	20.40		
pRIC4-BFDV-CP-OAS	Standard	1 pg	20.33		
pRIC4-BFDV-CP-OAS	Standard	0.1 pg	23.63	23.53	11,003.4823
pRIC4-BFDV-CP-OAS	Standard	0.1 pg	23.48		
pRIC4-BFDV-CP-OAS	Standard	0.1 pg	23.48		
pRIC4-BFDV-CP-OAS	Standard	0.01 pg	26.91	26.92	1100.34823
pRIC4-BFDV-CP-OAS	Standard	0.01 pg	26.92		
pRIC4-BFDV-CP-OAS	Standard	0.01 pg	26.92		
pRIC4-BFDV-CP-OAS	Standard	1 fg	28.79	28.78	110.034823
pRIC4-BFDV-CP-OAS	Standard	1 fg	28.96		
pRIC4-BFDV-CP-OAS	Standard	1 fg	28.61		
cDNA	Sample	A 1:100	13.78	13.88	566,945,200
cDNA	Sample	A 1:100	14.00		
cDNA	Sample	A 1:100	13.86		
cDNA	Sample	A 1:1000	17.08	17.06	710,799,200
cDNA	Sample	A 1:1000	17.05		
cDNA	Sample	A 1:1000	17.06		
cDNA	Sample	B 1:100	13.72	13.65	660,147,800
cDNA	Sample	B 1:100	13.65		
cDNA	Sample	B 1:100	13.57		
cDNA	Sample	B 1:1000	17.00	16.98	748,875,800
cDNA	Sample	B 1:1000	16.99		
cDNA	Sample	B 1:1000	16.96		
cDNA	Sample	C 1:100	13.93	13.85	576,893,100
cDNA	Sample	C 1:100	13.87		
cDNA	Sample	C 1:100	13.76		
cDNA	Sample	C 1:1000	16.51	16.50	1,024,206,000
cDNA	Sample	C 1:1000	16.53		
cDNA	Sample	C 1:1000	16.47		
