# Supplementary material for: Immunogenicity of DNA, mRNA and Subunit Vaccines Against Beak and Feather Disease Virus
Source: Vaccines (Basel). 2025 Jul 17;13(7):762. doi: 10.3390/vaccines13070762 (PMC12298428; doi:10.3390/vaccines13070762)
Supplement: Supplementary file 1 [file vaccines-13-00762-s001.zip › vaccines-3703131-supplementary.pdf]

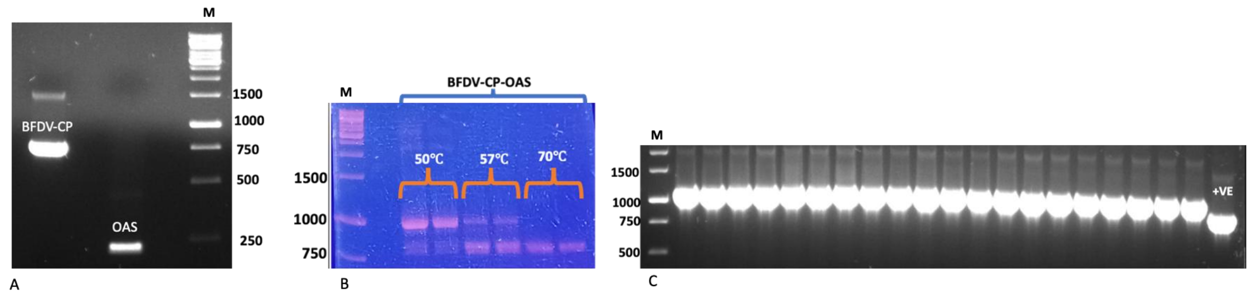

Figure S1. Creating the pRIC4-BFDV-CP-OAS construct. Gel electrophoresis images of PCR amplification for the BFDV-CP gene and OAS sequence (**A**), and gradient-PCR image for the fusion-PCR of the two fragments (**B**). Colony PCR screen for the pRIC4-BFDV-CP-OAS *E. coli* transformants (**C**). **M**: GeneRuler ™ 1 kb DNA Ladder in bp (Thermo Fisher). **+VE** : PCR positive control.

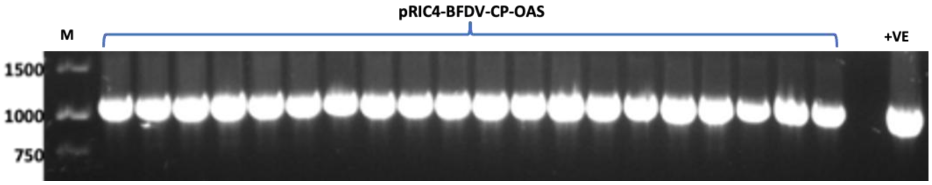

Figure S2. Confirmation of recombinant *Agrobacterium*. Gel electrophoresis image for colony PCR screening of GV3101::pMP90RK-pRIC4-BFDV-CP-OAS transformants. **Lane 1**: GeneRuler ™ 1 kb DNA Ladder in bp (Thermo Fisher). **+VE**: PCR positive control.

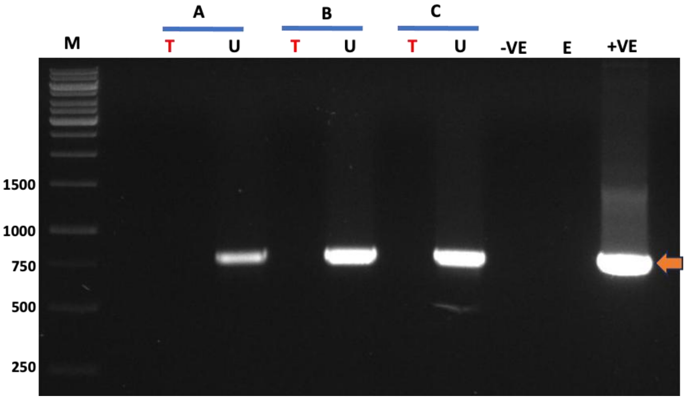

Figure S3. PCR to confirm DNase treatment of RNA samples. RNA extracted from 140 µL TMV particles sample (**A**), 280 µL sample (**B**) and 560 µL sample (**C**). GeneRuler ™ 1 kb DNA Ladder in bp (**M**), RNA treated with DNase was used as PCR template (**T**), Untreated RNA was used as PCR template (**U**), No template reaction (**-VE**), Empty well (**E**), Plasmid DNA was used as PCR template (**+VE**).

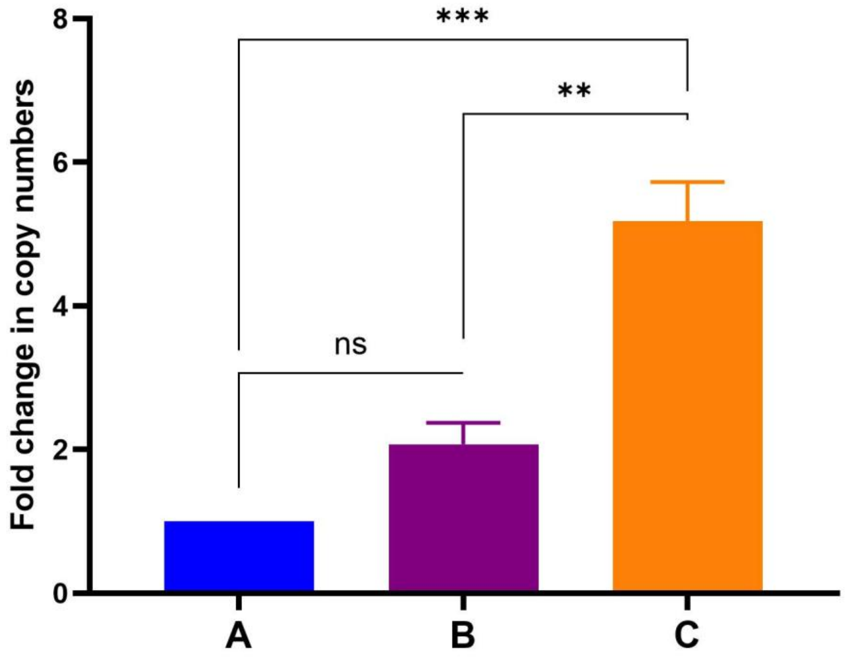

Figure S4. Fold change in BFDV-CP gene copy numbers between samples A, B and C. Average Cq values of three individual experiments in triplicates with RNA samples from three different extractions. The ΔΔCT method was used to quantify the differential expression of BFDV CP in samples B and C, using sample A as the reference control. Error bars represent standard error of means. Statistical significance was calculated using the Ordinary one-way ANOVA and *p* values were calculated based on the Tukey's multiple comparisons test with 95% Cl. Asterisk (*) denotes statistical significance. The mRNA copies in sample B were not significantly higher (p=0.1674) compared to sample A control (denoted by ns), while sample C mRNA copies were significantly higher (p=0.0004 and p=0.0021) compared to both A and B samples, respectively (denoted by *** and **).

Table S1: Additional information of the African grey parrot chicks that were used in the trial

Ring No.	Weight	BFDV PCR test tube No.	BFDV PCR test result	Vaccine Group
8467	480	12	Negative	BPFDV -CP Vaccine
8481	500	14	Negative	BPFDV -CP Vaccine
8475	528	3	Negative	BPFDV -CP Vaccine
8463	538	17	Negative	BPFDV -CP Vaccine
8479	555	6	Negative	BPFDV -CP Vaccine
8464	451	18	Negative	DNA Vaccine
8473	500	5	Negative	DNA Vaccine
8465	520	20	Negative	DNA Vaccine
8480	538	13	Negative	DNA Vaccine
8466	554	16	Negative	DNA Vaccine
8470	490	7	Negative	mRNA Vaccine
8468	510	2	Negative	mRNA Vaccine
8472	532	11	Negative	mRNA Vaccine
8477	539	1	Negative	mRNA Vaccine
8474	560	15	Negative	mRNA Vaccine
8476	500	4	Negative	Negative Control
8478	519	8	Negative	Negative Control
8469	537	10	Negative	Negative Control
8461	540	19	Negative	Negative Control
8471	561	9	Negative	Negative Control

Table S2. Additional results confirming successful encapsidation of BFDV cp mRNA within TMV particles.

Target	Sample Type	Sample	Cq	Average Cq	Absolute Copies
pRIC4-BFDV-CP-OAS	Standard	1ng	9.735284842	9.549726706	110034823
pRIC4-BFDV-CP-OAS	Standard	1ng	9.420556335		
pRIC4-BFDV-CP-OAS	Standard	1ng	9.493338939		
pRIC4-BFDV-CP-OAS	Standard	0.1ng	13.25920164	13.1581432	11003482.3
pRIC4-BFDV-CP-OAS	Standard	0.1ng	13.058731		
pRIC4-BFDV-CP-OAS	Standard	0.1ng	13.15649697		
pRIC4-BFDV-CP-OAS	Standard	10pg	16.86986954	16.8673498	1100348.23
pRIC4-BFDV-CP-OAS	Standard	10pg	16.86545465		
pRIC4-BFDV-CP-OAS	Standard	10pg	16.86672522		
pRIC4-BFDV-CP-OAS	Standard	1pg	20.49859989	20.64798394	110034.823
pRIC4-BFDV-CP-OAS	Standard	1pg	20.57208417		
pRIC4-BFDV-CP-OAS	Standard	1pg	20.87326776		
pRIC4-BFDV-CP-OAS	Standard	0.1pg	24.15818936	24.03707191	11003.4823
pRIC4-BFDV-CP-OAS	Standard	0.1pg			
pRIC4-BFDV-CP-OAS	Standard	0.1pg	23.91595446		
pRIC4-BFDV-CP-OAS	Standard	0.01pg	27.57690685	27.57732894	1100.34823
pRIC4-BFDV-CP-OAS	Standard	0.01pg	27.69154373		
pRIC4-BFDV-CP-OAS	Standard	0.01pg	27.46353625		
pRIC4-BFDV-CP-OAS	Standard	1fg	30.48137245	31.09433661	110.034823
pRIC4-BFDV-CP-OAS	Standard	1fg	31.58357643		
pRIC4-BFDV-CP-OAS	Standard	1fg	31.21806094		
cDNA	Sample	BFDV-CP-OAS 1:100	20.48252706	20.45648896	10894060
cDNA	Sample	BFDV-CP-OAS 1:100	20.48179228		
cDNA	Sample	BFDV-CP-OAS 1:100	20.40514754		
